# Non-Invasive Diagnosis of Endometriosis by Questionnaires in Patients Using Contraception

**DOI:** 10.3390/jcm15010030

**Published:** 2025-12-20

**Authors:** Felix Zeppernick, Samira Balimuttajjo, Christian Schorr, Florian Sibelius, Manuela Schuler, Sebastian Harth, Sarah Seeger, Anna Löffelmann, Muhammad A. Riaz, Ivo Meinhold-Heerlein, Lutz Konrad

**Affiliations:** 1 Institute of Gynecology and Obstetrics, Faculty of Medicine, Justus Liebig University Giessen, Feulgenstr. 10-12, 35392 Giessen, Germany; felix.zeppernick@gyn.med.uni-giessen.de (F.Z.); samira.s.balimuttajjo@med.uni-giessen.de (S.B.); anna.loeffelmann@paediat.med.uni-giessen.de (A.L.); muhammad.a.riaz@gyn.med.uni-giessen.de (M.A.R.); ivo.meinhold-heerlein@gyn.med.uni-giessen.de (I.M.-H.); 2 Deutsches Forschungszentrum für Künstliche Intelligenz GmbH, DFKI Saarland Informatics Campus D3.2, 66123 Saarbrücken, Germany; christian.schorr@dfki.de (C.S.); florian.sibelius@dfki.de (F.S.); manuela.schuler@dfki.de (M.S.); 3 Department of Diagnostic and Interventional Radiology, Faculty of Medicine, Justus Liebig University Giessen, 35390 Giessen, Germany; sebastian.harth@radiol.med.uni-giessen.de

**Keywords:** endometriosis, pelvic pain, neuropathic pain, PainDETECT, prediction models, pre-operative diagnosis, questionnaire, contraception

## Abstract

**Background/Objectives:** The assessment of endometriosis (EMS)-associated pain is important, but only very few studies address the potential use of questionnaires for non-invasive prediction of the disease. **Methods:** In a prospective observational study from 2016 to 2024 with patients (n = 228) using hormonal contraception, all women with suspected EMS answered two questionnaires and were examined physically and with transvaginal ultrasound (TVUS). If deep infiltrating EMS (DIE) was suspected, magnetic resonance imaging (MRI) was performed. EMS diagnosis was confirmed by histological examination. Statistical analysis was mainly performed using 2 × 2 contingency tables. The decision tree was created manually. **Results:** The mean numerical rating scales (NRSs) of EMS-positive compared to EMS-negative patients were ~4-fold higher (4.45 and 1.15, respectively). Patients with EMS have, significantly, ~3 times more significant parameters compared to patients without EMS (18.5 and 5.9, respectively). In combination with dysuria and lightning-like pain, this resulted in very good prediction. A decision tree yielded a sensitivity of 0.924, a specificity of 0.917, a positive predictive value (PPV) of 0.924, a negative predictive value (NPV) of 0.917, and a positive likelihood ratio of 11.2, indicating a very good diagnostic test. There is no typical endometriosis pain, but various pain patterns are predictive of EMS. **Conclusions:** Thus, a reliable non-invasive EMS diagnosis by questionnaires is possible and could reduce the delay in the detection of EMS.

## 1. Introduction

Endometriosis (EMS) is a disease defined by the implantation and growth of endometrial tissue outside the uterine cavity [1]. The disease can last for decades, mainly characterized by pain, usually in the pelvic area, and infertility [2]. Pain is defined as “An unpleasant sensory and emotional experience associated with, or resembling that associated with, actual or potential tissue damage” [3].

The most frequently reported types of EMS pain are dysmenorrhea, chronic pelvic pain (CPP), non-menstrual CPP, and dyspareunia [1], with dysmenorrhea being the most common pain symptom and usually causing the greatest perception of pain [4,5]. Dysmenorrhea is characterized by frequent and severe menstrual cramps and pain during menstruation [6] and more women with EMS versus without EMS had menstrual pain/cramping and non-menstrual pelvic pain/cramping [7]. Remarkably, pain can also be described using adjectives, though this is rarely done in EMS. In our recent prospective study in women not using contraception, we found that a significantly greater proportion of women with EMS versus without EMS, but without contraception, reported their EMS-associated pain as cramping, tearing, pulling, stabbing, pulsating, burning, and touch-sensitive [8]. In contrast, Ballard et al. [9] reported that gnawing and throbbing were more common in women with EMS, but not cramping or pulling.

Interestingly, despite revealing a very large number of pain parameters not previously described for EMS, we found no EMS-specific pain, but more significant parameters in EMS cases [8]. Similarly, an increased likelihood of EMS with a greater number of symptoms was also reported by another study (85-fold ≥ 7 symptoms) [10]. Furthermore, women with EMS reported the highest chronic/cyclic pain and significantly greater dyspareunia, dysmenorrhea, and dyschezia compared to women without EMS [11]. A large study with the Endometriosis Health Profile-30 questionnaire found that women with EMS compared to without EMS experienced more menstrual pain, abdominal pain unrelated to menses, defecation pain, irregular bleeding, and bowel irritation (constipation/diarrhea) [12]. Another study with a questionnaire revealed that dysmenorrhea, dyspareunia, dysuria, lower back pain, pelvic pain other than during menses, rectal pain, and pain at ovulation or intercourse are strongly linked to EMS [13]. Similarly, Fourquet et al. [14] also reported high pain values among EMS patients including dysmenorrhea, dyspareunia, infertility, dyschezia, and dysuria. However, the study did not specify the use or non-use of contraceptives. In an online survey with several different questionnaires, severity and duration of menstrual pain, bloating, nausea, and widespread pain sites were found to be significant predictors of EMS [15]. In summary, all studies agree that a constellation of EMS-related symptoms, rather than a single parameter, is relevant for the diagnosis of the disease [2].

To improve EMS diagnostics, there is still a need for a non-invasive test that does not necessarily have to be based on blood, urine, or saliva measurements. Several studies with questionnaires have shown very good predictive values [6,13,16,17]. More recently, similar results have been obtained using machine learning [18,19,20]. At the beginning of our study on the diagnosis of EMS with questionnaires, we found that the pain of patients with/without contraceptives (OC) differed significantly in terms of quantity, pain intensity, and pain patterns [8]. For example, the ratio of significant parameters for women without contraception without EMS vs. with EMS is 1:2 [8], and with contraception 1:3. Previous studies using questionnaires have not sufficiently acknowledged these differences. In this study, the primary aim was to identify the pain characteristics of EMS patients with OCs and to use them for non-invasive prediction/diagnosis of EMS. For this purpose, a decision tree was generated. Furthermore, pain patterns of EMS patients were identified and used for non-invasive prediction/diagnosis of the disease. They are intended to assist in the diagnosis of all sorts of EMS, but also in cases with peritoneal EMS, which is not easy to diagnose, and to reduce diagnostic delays and doctor hopping, and to enable patient self-monitoring of the disease.

## 2. Material and Methods

### 2.1. Participants

This prospective observational study was conducted at the University Hospital Giessen, Germany, from 1 September 2016 until 29 February 2024. The local ethics committee approved this study (No. 95/09, July 2009) and all patients signed their informed consent. The inclusion criteria were as follows: all women with pelvic pain (menstruation-associated pain) and infertility problems and all women who were referred to our endometriosis centre by their general practitioners. The exclusion criteria were as follows: cases with cancer, pregnant women, women who have had a pelvic laparoscopy during the last 6 months prior to visiting our centre, and women who have had a hysterectomy, bladder infections, or suffered from nutcracker syndrome. Every patient who was referred to the clinic was included in this study. As the proportion of women who are highly likely to have EMS predominates in clinics, volunteers (n = 20) were included to avoid bias. All women were examined by physical examination, palpation, and TVUS to exclude EMS. If DIE was suspected, an MRI was performed. Only patients with suspected EMS by medical examination were operated on and the tissue subsequently examined histologically. DIE was classified intraoperatively with the ENZIAN [21] and #ENZIAN score [22].

Women with a negative medical examination, including TVUS for all and MRI for n = 36 women with suspected DIE, were assigned to the control group.

### 2.2. Main Variables

We used a validated questionnaire recently developed by us [8] and additionally the PainDETECT questionnaire to investigate neuropathic pain [23,24]. The questionnaire was optimized by experienced EMS specialists in collaboration with EMS patients and volunteers. The details to both questionnaires and methods can be found in our recent manuscript [8]. Both questionnaires were given to patients before the clinical examination, and, if needed, healthcare professionals and/or medical students helped patients to complete them. Even though there was sometimes missing data, it was still possible to evaluate nearly all patients and to make an unequivocal prediction. This is presumably due to the very large number of parameters that were asked, so that a single missing parameter was not decisive.

The questionnaires, clinical examinations, surgical findings, and histological diagnoses were collected for data analysis. The data was anonymized and presented in tabular form in Excel. Diagnosis of EMS by histological examination was possible in 99.16% of women with EMS. In one patient the lesions were coagulated intraoperatively. As both the medical examination and the imaging procedures were clearly positive, this patient was included in this study. In total we found the following EMS cases: 9 ovarian (OV), 15 OV/peritoneal (PE) EMS, 60 PE (one case with EMS in the paracolic gutter), 1 OV/DIE, 9 PE/DIE (including one case with EMS in the paracolic gutter), 13 DIE, and 7 OV/PE/DIE.

### 2.3. Statistics

All parameters were evaluated with 2 × 2 contingency tables and Fisher’s exact test. The mean values ± SD were calculated for the NRSs, and comparisons between the groups were performed using the nonparametric Mann–Whitney test. The sum VAS (ΣVAS) was derived from the individual VAS values of the parameters dysmenorrhea, dyspareunia, dysuria, dyschezia, CPP, pain now, strongest pain in 4 weeks, and average pain. The significant parameters (SPs) that contribute to the sum of SPs (ΣSP) are marked with an asterisk in all tables. Cut-offs were calculated with a receiver operating characteristics (ROC) curve and area under the curve (AUC) with a 95% confidence interval (CI). The prediction models were hand-crafted. For all models, the values for negative predictive values (NPVs), positive predictive values (PPVs), sensitivity, specificity, odds ratio (OR), and relative risk (RR) were calculated as published [8]. The odds ratio as a diagnostic odds ratio (DOR) was used to facilitate the comparison of diagnostic tests [25]. *p* values ≤ 0.05 were considered to be statistically significant.

## 3. Results

### 3.1. Study Sample

No correlations could be found for age, BMI, age at menarche, smoking habits, and allergies, except for the intake of analgesics between women without or with EMS (Table 1). Most patients with or without EMS utilized dienogest (DNG) and significantly less frequently levonorgestrel (LNG) or desogestrel (DSG) (Table 1).

### 3.2. “Classical” EMS Parameters

The majority of both patient groups suffered from dysmenorrhea, but more women with EMS had dysmenorrhea (89/119 = 74.8%) compared to women without the disease (48/109 = 44.0%), and a cutoff at NRS > 3 was even more suitable for discrimination (Table 2).

Despite contraception, the pain intensity of dysmenorrhea was significantly ~2 times higher in women with EMS than in women without EMS. Similarly, more women with EMS suffered from CPP, with clearly higher NRS scores than women without EMS (Table 2). Remarkably, significantly more women with EMS suffered from painful urination (35.3%) than women without EMS (0.9%). The intensity of pain also differed significantly (~31-fold) between the two groups. This was an important indication of the relevance of dysuria as a differentiating criterion between both groups (Table 2).

A significantly greater proportion of women with EMS reported pain and had a ~4.2-fold higher NRS score at defecation than women without EMS (Table 2). Similarly, a significantly higher proportion of women with EMS suffered from constipation and diarrhea than women without EMS (Table 2). The pain intensity in women with EMS during sexual intercourse was notably ~3-fold higher compared to women without the condition (Table 2).

### 3.3. Pain Descriptions with Adjectives

Nearly all adjectives for pain description showed a highly significant correlation with EMS, with the highest values for cramping and pulling, suggesting that these may primarily denote mechanical pain. Furthermore, it was notable that stinging/stabbing and flashing/lightning-like pain also had very high scores (Table 3).

### 3.4. PainDETECT and Pain Localization

The PainDETECT indicated highly significant relationships between the three pain scores, current pain, most severe pain, and average pain during the last 4 weeks, with EMS, both in the proportions of patients and in pain intensity (Table 4). Pain was localized in the lower back, lower abdomen, upper abdomen, thighs/legs, vagina, and hips/groin by a significantly higher proportion of patients with EMS than patients without EMS. Remarkably, the pain pattern was also different and, except for persistent pain, with mild fluctuations. The final scores of neuropathic pain were significantly higher in women with EMS compared to those without EMS, and more women (8.4%) with EMS experienced neuropathic pain compared to women without EMS (1.8%) (Table 5).

The final scores of neuropathic pain were significantly higher in women with EMS compared to those without EMS, and more women (8.4%) with EMS experienced neuropathic pain compared to women without EMS (1.8%) (Table 5). We used a different cut-off (0–3 vs. >3) of the final score to better discriminate between women with and without EMS.

### 3.5. Decision Tree

We found that women with EMS experienced more severe pain compared to those without EMS. We summarized this with the ΣVAS score and found ~4-fold higher values in women with EMS versus those without EMS. A cut-off of 8.5 showed a very high degree of discrimination between the two patient groups (Table 6).

**Figure 1:**
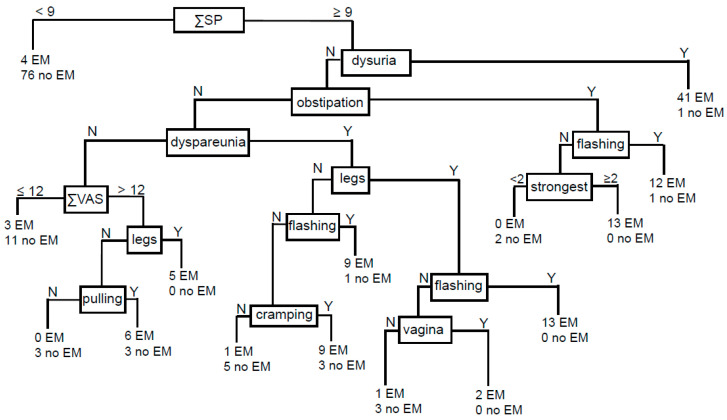
The sum of significant parameters (ΣSP) with a cutoff at 9 was used as the root of the decision tree. Similarly, the sum of VAS (ΣVAS) with a cut-off of 12 was suitable for prediction. The “classical” endometriosis pain parameters dysuria, dyspareunia, and obstipation were good predictors. Pain in the thigs/legs and vagina denoted pain location, whereas cramping and pulling were the best discriminating adjectives. From the PainDETECT questionnaire we used the strongest pain in 4 weeks and lightning/flashing pain for prediction. In summary, 11 significant parameters were useful. N, no; Y, Yes; Sp, significant parameters; strongest, strongest pain/4 wks; EM, endometriosis; no EM, no endometriosis.

Similarly, women with EMS versus women without EMS have on average ~3 times as many SPs. Again, a cut-off of 8.5 is suitable for discrimination and for a moderate prediction of EMS (Table 6). However, when the ΣSPs were used as the root, the generated decision tree allowed for a substantial differentiation between women with EMS and without EMS (Figure 1). The positive likelihood ratio (LR+) was 11.13 and the LR- 0.83 (Table 6), which is in favour of convincing diagnostic evidence [26]. Both values together resulted in very high values for DOR (~136) and accuracy (92.1%).

## 4. Discussion

In this study, we analyzed a large number of predominantly pain parameters in patients suspected for EMS with contraception, similarly to our recent study with EMS patients without contraception [8]. In both studies, we found EMS-typical pain patterns, significantly more pain parameters, and stronger pain in women with EMS compared to women without EMS. The generated decision trees resulted in an OD/DOR of 135.8 for the hand-crafted decision tree, suggesting that questionnaires could be an attractive tool for preoperative and non-invasive EMS diagnosis.

We found no associations of the general parameters including age, BMI, smoking, menstruation duration, cycle duration, fertility, and allergies with EMS; however, the significance of these parameters for EMS is controversial [7,17,27,28,29,30]. Furthermore, as we have already shown in our previous study, these parameters are not useful for the prediction of EMS [8]. In contrast, a significantly higher proportion of women with EMS (85.7%) used analgesics during menstruation than women without EMS (36.7%). Notably, we found similar values in women without/with EMS but without contraception [8], suggesting that contraception may reduce analgesic use, but only in women without EMS. Our values are similar to a previous report [16]; however, the use or non-use of hormonal contraception was not distinguished.

Our results showed that the “classical” EMS-associated parameters dysmenorrhea, CPP, dysuria, dyschezia, dyspareunia, constipation, and diarrhea are highly significantly associated with EMS, comparable to recent observations [2,8,11]. Nearly all patients with EMS showed significantly higher NRS scores compared to women without EMS. Interestingly, ~39 times more women with EMS (35.3%) suffered from dysuria compared to very few women without EMS (0.9%). We had recently found similarly high values (32.2%) for the group of women with EMS but without contraception [8]. Similar results were described in other studies, but these did not distinguish between contraceptive use and non-use [16] or did not include a control group [14]. Due to the very high discriminatory power of dysuria for a subgroup of EMS patients, this parameter was used for prediction (Figure 1). It is therefore important to ensure that dysuria is not caused by cystitis.

When differentiating the various pain qualities using adjectives, a significantly greater proportion of women with EMS compared to those without EMS described their pain as cramping, tearing, pulling, stabbing, pulsating, sensitive to touch, burning, pressing, cold/warm, and flashing. We recently found similar descriptions in women with EMS but without contraception [8]. Cramps during [6] and outside menstruation have also been described in women with EMS versus those without EMS in other studies [7].

The PainDETECT questionnaire showed a higher prevalence of neuropathic pain in patients with EMS (40%) [31] or with CPP (26%) [32]. In contrast, we identified a lower percentage (8.4%), similar to our recent study of EMS patients without contraception [8]. Nevertheless, we were able to confirm that more women with EMS suffered from neuropathic pain than women without EMS. At this point, we would like to briefly touch upon whether neuropathic pain in EMS patients is permanent or transient. In most cases, neuropathic pain decreases or disappears after surgery, an important indication being, for example, the very low reoperation rates (0.4–1.4%) after hysterectomies in EMS cases with or without preservation of the ovaries [33]. Thus, it seems highly likely that neuropathic pain is in most patients transient.

A higher proportion of women with EMS compared to women without EMS reported more severe current pain, more severe pain in the last 4 weeks, and more severe average pain, as we have already reported for women with EMS but without contraception [8]. The occurrence of pain in the lower and upper abdomen, lumbar spine, hips/groin, thighs/legs, vagina/mons pubis, and gluteal region was also more common in cases with EMS than in cases without EMS. Similar results were also observed in our study of EMS cases without contraception [8]. Spread of EMS pain to the thighs/legs is common and has been described in 51.5% (48/94) [34] or 33.9% (60/177) [8] of women with EMS. This is believed to result from the fusion of uterine tissue with the sciatic nerve, persistent inflammation, and neural discharge, as shown in an animal model [35]. Another possibility would be EMS of the obturator nerve, but this is extremely rare and is characterized by pain in the inner thigh [36].

In summary, as previously described for EMS patients without contraception [8], we again observed that no single pain parameter was significant as a prognostic factor, which is consistent with a previously published review [2]. Thus, we assume with a high probability that pain patterns are typical for EMS. We again found an increased likelihood of EMS with an increased number of SPs, this time ~3-fold compared to women without EMS. Based on these results, we created a decision tree for EMS prediction (Figure 1). It is noteworthy that the number of significant parameters was again useful as the root of the tree, as previously published for cases with EMS but without contraception [8]. The decision tree yielded a high sensitivity of 0.924, a specificity of 0.917, an LR+ of 11.2, and an LR− of 0.083, resulting in an OD/DOR of ~136. For comparison, we calculated the OD/DOR values from a recent review [37], and obtained an OD/DOR of 9.5 for the bimanual examination (sensitivity 0.76, specificity 0.75), an OD/DOR of 58.9 for TVUS (sensitivity 0.79, specificity 0.94), and an OD/DOR of 52.4 for MRI (sensitivity 0.94, specificity 0.77). A clinically useful diagnostic test for EMS was postulated to have a sensitivity of 0.94 and a specificity of 0.79 [38], resulting in an OD/DOR of 58.9. Not only is our prediction significantly higher than these values, but it should also be noted that of the clinical examination methods, only TVUS fulfils these criteria [38]. In addition, it is known that TVUS and MRI are not very good for the detection of peritoneal EMS [37,39], while our questionnaire is able to detect all three EMS entities: ovarian, peritoneal, and DIE. In a study by Ricci et al. [16], they found a sensitivity of 90% and a specificity of 75%, which results in a DOR/OR of 27. Bendifallah et al. [18] calculated a sensitivity of 93% and specificity of 92% with XGB, resulting in a DOR/OR of 154.5 comparable to our results but with a considerably higher number of cases (n = 1734). In our opinion this underscores that the quality of the questionnaire might be more important than the number of cases. Of note, Goldstein & Cohen [19] obtained the highest values of 93.2% sensitivity and 94.6% specificity with Ada Boost, resulting in a DOR/OR of 210. However, they acknowledge that in their study with n = 886 questionnaire responders, n = 412 were without an EMS diagnosis. It should be noted also that in the three machine learning studies published on EMS prediction, none used contraceptive use as a discriminating criterion. Nevertheless, questions concerning periods were included as significant parameters in the prediction [18,19,20]. Since we found a clear difference between the groups with and without contraception, we have reservations about EMS predictions that do not mention this.

### Strength, Weaknesses, and Implications

The strength of our study is the very comprehensive recording of many parameters, especially pain. The highly significant non-invasive prediction of EMS with questionnaires cannot only be carried out by patients at home at their leisure, but is free of charge and at least as good if not better than medical examination. Nevertheless, for validation of the questionnaire, all women underwent a medical examination and if necessary an operation. The questionnaire was optimized several times and validated.

Despite a standardized and very careful diagnostic laparoscopy including inspection of the diaphragm/upper abdomen, it cannot be ruled out that foci were overlooked during operation. In addition, women with positive results in the questionnaire but negative results in the medical examination were not operated on for ethical reasons.

This study has the following weaknesses: (1) a lack of surgical confirmation in the control subjects, (2) a single-centre design, (3) a lack of external validation, (4) selection bias due to recruitment in clinics, (5) unclear transferability to primary care, and (6) potential bias in completing the questionnaire. However, to the best of our knowledge all previously published studies on questionnaires have more or less these limitations. As can be seen from our missing data, the patients answered almost all questions, so that the extent of the bias regarding completion of the questionnaire appears negligible.

Nevertheless, the prognostic model presented is well suited for the non-invasive diagnosis of EMS, but can also be used to monitor the disease before and after surgery with or without drug therapy or drug therapy alone. Another strength is that the questionnaires also enabled many cases of peritoneal EMS to be correctly diagnosed, which is not so easy in clinical practice. In the future, patients can be categorized in advance into those who need immediate help and those who only need to be monitored with or without therapy. This would save time and costs, prevent unnecessary doctor visits, and avoid unnecessary operations, therapies, or overdiagnosis. The diagnosis/prediction of EMS by general practitioners or as an aid for patients will significantly reduce the well known very long diagnostic delay.

## 5. Conclusions

In this study, we have shown that pain patterns have a high predictive power for EMS. In contrast to women without EMS, women with EMS have more pain and more severe pain. Interestingly, a very high number of cases described their pain as cramping or pulling, indicating muscle contractions in the pelvis, presumably mainly caused by the uterus in conjunction with the endometriotic lesions. Together with our former study we now can distinguish and recognize pre-operatively and non-invasively endometriosis in women with and without contraceptives with our questionnaire. Doctors should therefore always consider endometriosis as a possible cause of severe menstrual pain and treat it accordingly.

## Figures and Tables

**Table 1:** Demographics of patients with and without endometriosis.

	No EMS n = 109	MD	EMS n = 119	MD	*p* Value
Age (yrs), mean (SD)	31.0 (8.3)	0	30.8 (6.8)	0	0.810
BMI, mean (SD)	24.3 (6.0)	0	24.7 (4.8)	1	0.1082
Age menarche, mean (SD)	13.0 (1.6)	0	12.7 (1.6)	0	0.1066
Smoker (%)	19 (17.4)	0	32 (26.9)	0	0.111
Allergies (%)	52 (47.7)	0	66 (55.5)	0	0.288
Cycle duration in days (SD)	26.3 (3.6)	74	27.6 (3.5)	82	n.c.
Menses duration in days (SD)	5.0 (1.4)	72	5.7 (2.6)	80	n.c.
Use of analgesics (%) *	40 (36.7)	0	102 (85.7)	0	0.0001
	No	Yes	No	Yes	
Fertility	15	30	16	42	n.c.
Nulligravid/nulliparous	64	0	61	0	
OC/contraception	109	6	119	5	
DNG ± E (%)	42 (38.5)		64 (53.8)		
LNG ± E (%)	25 (23.0)		20 (16.8)		
DSG ± E (%)	21 (19.3)		20 (16.8)		
E + others (%)	7 (6.4)		7 (5.9)		
Other OCs (%)	8 (7.3)		3 (2.5)		

* Counted as significant parameters; EMS, endometriosis; MD, missing data; BMI, body mass index; yrs, years; SD, standard deviation; LNG, levonorgestrel; DNG, Dienogest; DSG, desogestrel; n.c., not calculated; *p* values were calculated by Fisher’s exact test (smoker, allergies, fertility) or Mann–Whitney (all the others) where appropriate.

**Table 2:** “Classical” pain parameters of patients with and without endometriosis.

	No EMS n = 109		EMS n = 119		*p* Value
	No	Yes	No	Yes	
Dysmenorrhea (PP) *	61	48	30	89	0.0001
PP, NRS > 3 *	72	37	31	88	0.0001
PP, NRS score (SD)	2.9 (3.8)		(3.9)		0.0001
PP before menses (%)	107 (98.2)	2	115 (96.6)	4	0.6852
PP during menses (%)	105 (96.3)	4	111 (93.3)	8	0.3805
PP before/during menses (%) *	93 (85.3)	16	76 (63.9)	43	0.0003
PP before/during/after menses (%)	101 (92.7)	8	100 (84.0)	19	0.0634
PP after menses (%)	108 (99.1)	1	118 (99.2)	1	1.0
PP during/after menses (%)	109 (100)	0	118 (99.2)	1	n.p.
PP before/after menses (%)	108 (99.1)	1	118 (99.2)	1	1.0
PP time interval (MD)		5		11	
Chronic pelvic pain (CPP) *	94	15	35	84	0.0001
CPP, NRS score (SD)	0.78 (2.2)		4.5 (3.6)		0.0001 ^$^
Dysuria (painful urination) *	108	1	77	42	0.0001
Dysuria, NRS score (SD)	0.05 (0.5)		1.53 (2.5)		0.0001
Dyschezia (painful defecation) *	95	14	51	68	0.0001
Dyschezia, NRS score (SD)	0.77 (2.2)		3.2 (3.3)		0.0001
Dyspareunia (painful intercourse) *	78	31	27	92	0.0001
Dyspareunia, NRS score (SD)	1.43 (2.7)		4.5 (3.1)		0.0001
Obstipation *	98	11	68	51	0.0001
Diarrhea *	83	26	53	66	0.0001

* Counted as significant parameter; EMS, endometriosis; SD, standard deviation; ^$^ 1 missing data (MD); *p* values calculated by Fisher’s exact test or Mann–Whitney (NRS scores) where appropriate; n.p., not possible due to one variable being zero.

**Table 3:** Pain sensations of patients with and without endometriosis.

	No EMS (n = 109)		EMS (n = 119)		*p* Value
	No	Yes	No	Yes	
Cramping *	66	43	22	97	0.0001
Tearing *	95	14	65	54	0.0001
Pulling *	74	35	32	87	0.0001
Stinging *	80	29	46	73	0.0001
Pulsatile *	99	10	82	37	0.0001
Touch-sensitive *	87	22	64	55	0.0001
Burning	101	8	95	24	0.007
Pressing *	95	14	61	58	0.0001
Diffuse	102	7	104	15	0.1232
Cold/warmth *	108	1	100	19	0.0001
Flashing *	102	7	52	67	0.0001

* Counted as significant parameter; EMS, endometriosis; no missing data found; *p* values were calculated by Fisher’s exact test.

**Table 4:** Pain characteristics and localization with the PainDETECT.

	No EMS n = 109		EMS n = 119		*p* Value
	No	Yes	No	Yes	
Pain now *	94	15	34	85	0.0001
Pain now, NRS (SD)	0.48 (1.46)		2.82 (2.72)		0.0001
Strongest pain/4 wks *	74	35	3	116	0.0001
Strongest pain/4 wks, NRS (SD)	1.8 (3.1)		7.7 (2.5)		0.0001
Average pain *	80 (MD 1)	28	4	115	0.0001
Average pain, NRS (SD)	1.07 (2.25)		5.08 (2.3)		0.0001
Lower abdomen *	76	33	7	112	0.0001
Lumbar spine *	83	26	44	75	0.0001
Thighs/legs *	103	6	67	52	0.0001
Hips/groins *	101	8	66	53	0.0001
Upper abdomen *	104	5	101	18	0.0085
Vagina/mons pubis *	106	3	95	24	0.0001
Gluteal region *	109	0	112	7	0.0148
Pain course pattern					
Persistent pain with slight fluctuations	100 (MD 1)	8	106	13	0.4923
Persistent pain with pain attacks *	100 (MD 1)	8	73	46	0.0001
Pain attacks without pain between them *	95 (MD 1)	13	86	33	0.0046
Pain attacks with pain between them *	103 (MD 1)	5	97	22	0.0017

* Counted as significant parameter; EMS, endometriosis; SD, standard deviation; wks, weeks; MD, missing data; *p* values calculated by Fisher’s exact test or Mann–Whitney (NRS).

**Table 5:** Neuropathic pain in women with and without EMS.

	No EMS n = 109		EMS n = 119		*p* Value
	No	Yes	No	Yes	
Final score (neuropathic pain)					
Neg 0–12 (%)	105 (96.3)		86 (72.3)		0.0001
Unclear 13–18 (%)	2 (1.8)		23 (19.3)		0.0001
Pos 19–38 (%)	2 (1.8)		10 (8.4)		0.0358
Final score, mean (SD)	2.3 (4.6)		10 (5.7)		0.0001
0 (%) vs. 1–38	72 (62.1)	37	5 (4.2)	114	0.0001
0–3 (%) vs. 4–38 *	83 (76.1)	26	14 (11.8)	105	0.0001

* Counted as significant parameter; EMS, endometriosis; SD, standard deviation; wks, weeks; MD, missing data; *p* values calculated by Fisher’s exact test or Mann–Whitney (NRS).

**Table 6:** Sum of VAS scores, sum of significant parameters, and decision tree analysis.

	No EMS (n = 109)		EMS (n = 119)		*p* Value
	No	Yes	No	Yes	
ΣVAS Score, mean (SD)	5.13 (5.94)		19.2 (6.58)		0.0001
Cut-off of 8.5 *	86	23	17	102	0.0001
ΣSP (SD)	5.98 (5.94)		18.88 (4.38)		0.0001
Cut-off of 8.5 *	76	33	4	115	0.0001
PPV (95% CI)	0.777 (0.703–0.837)				
NPV (95% CI)	0.950 (0.878–0.980)				
Sensitivity (95% CI)	0.966 (0.917–0.987)				
Specificity (95% CI)	0.697 (0.606–0.776)				
Odds ratio (95% CI)	66.21 (22.75–175.8)				
Relative risk (95% CI)	15.54 (6.36–39.75)				
Decision tree	100	9	9	110	0.0001
PPV (95% CI)	0.924 (0.863–0.960)				
NPV (95% CI)	0.917 (0.851–0.956)				
Sensitivity (95% CI)	0.924 (0.917–0.987)				
Specificity (95% CI)	0.917 (0.851–0.956)				
Odds ratio (95% CI)	135.8 (49.87–236.5)				
Relative risk (95% CI)	11.20 (6.167–21.03)				
LR+	11.13 (5.98–20.98)				
LR−	0.083 (0.041–0.155)				

* Counted as significant parameter (SP); EMS, endometriosis; PPV, positive predictive value; NPV, negative predictive value; CI, confidence interval; SD, standard deviation; LR+, positive likelihood ratio; LR−, negative likelihood ratio; *p* values were calculated by Fisher’s exact test.

## Data Availability

The data is available from the corresponding author upon request.
